# Quantifying the Renal Impact of [^177^Lu]Lu-PSMA-617: A Longitudinal Analysis of Real-World Data from the LUMEN Registry

**DOI:** 10.2967/jnumed.125.271077

**Published:** 2026-07

**Authors:** Thomas Büttner, Christian Hoffmann, Markus Essler, Florian Gärtner, Barbara Kreppel, Jim Küppers, Manuel Ritter, Leander Fritzsche, Milka Marinova, Philipp Krausewitz

**Affiliations:** 1 Department of Urology and Pediatric Urology, University Hospital Bonn, Bonn, Germany; and; 2 Department of Nuclear Medicine, University Hospital Bonn, Bonn, Germany

**Keywords:** mCRPC, ^177^Lu, PSMA, kidney, eGFR

## Abstract

This study aimed to clarify the renal safety of [^177^Lu]Lu-PSMA-617 for metastatic castration-resistant prostate cancer (mCRPC), addressing emerging reports of nephrotoxicity. **Methods:** We retrospectively analyzed patients with mCRPC from the real-world LUMEN registry who received 3 or more cycles of [^177^Lu]Lu-PSMA-617. The endpoint was the change in estimated glomerular filtration rate (eGFR) over 24 mo, assessed using linear mixed-effects models. **Results:** Analysis of 261 patients revealed a modest average decline in eGFR of −0.15 mL/min/1.73 m^2^ per month (95% CI, −0.25 to −0.05; *P* = 0.005), translating to −1.80 mL/min/1.73 m^2^ per year (95% CI, −3.03 to −0.57). The rate of loss accelerated by approximately −0.1 per year per additional gigabecquerel of administered activity (95% CI, −0.18 to −0.01; *P* = 0.028). **Conclusion:** [^177^Lu]Lu-PSMA-617 is associated with a slight long-term average decline in eGFR. Although this is unlikely to be clinically relevant for late-stage mCRPC, the cumulative dose effect warrants careful monitoring in earlier disease stages.

## Visual Abstract

**Figure:**
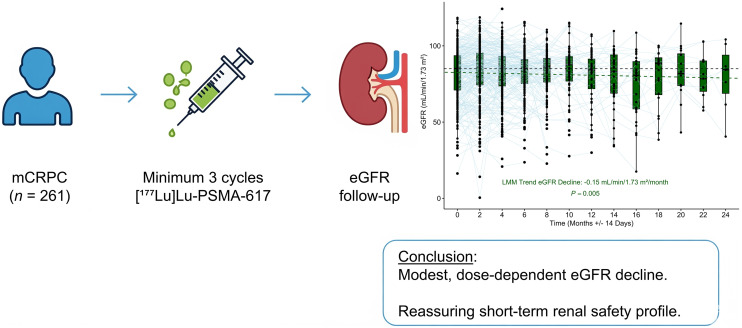

With the expanding clinical application of [^177^Lu]Lu-PSMA-617 for patients with metastatic castration-resistant prostate cancer (mCRPC), a comprehensive understanding of its long-term safety profile is essential. Although the pivotal VISION trial suggested minimal nephrotoxicity (5.3% all-grade and 0.2% grade ≥ 3 creatinine increase) with the use of [^177^Lu]Lu-PSMA-617 (1), recent postmarketing data suggest a potentially greater decline in renal function associated with this therapy (2,3). The discrepancy between trial conditions and clinical practice necessitates further investigation.

To address this discrepancy, we evaluated longitudinal renal function in a large, real-world cohort from the LUMEN registry. Our study aimed to quantify estimated glomerular filtration rate (eGFR) trajectories and identify risk factors for decline, with a specific focus on cumulative dose effects.

## MATERIALS AND METHODS

### Patient Cohort and Ethics

**FIGURE 1.**
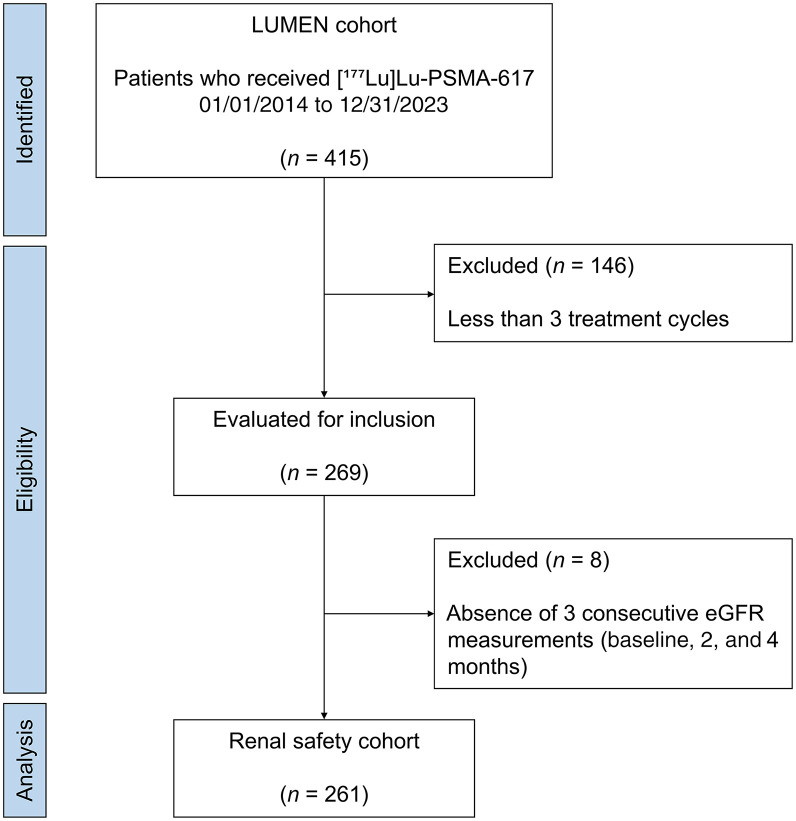
Cohort selection process.

This study used the LUMEN registry (German Clinical Trail Register ID DRKS00035087) to retrospectively identify all patients who received [^177^Lu]Lu-PSMA-617 at the University Hospital Bonn between 2014 and 2023. Inclusion required administration of at least 3 cycles of therapy, baseline serum creatinine, and at least 2 consecutive follow-up measurements (2-mo intervals ± 14 d) to ensure data quality for trajectory analysis (Fig. 1). The Ethics Commission at the Medical Faculty of the University of Bonn approved this retrospective study (vote ID 2024-19-BO), and the requirement to obtain informed consent was waived.

### Radiopharmaceutical Therapy

PSMA-617 (ABX GmbH) was radiolabeled with ^177^Lu as described previously (4). Patients received [^177^Lu]Lu-PSMA-617 as an intravenous bolus, followed by a 1,000-mL crystalloid infusion. Obstructive uropathy was excluded via renal scintigraphy before initiation of therapy. Cycles continued until disease progression or unacceptable toxicity. Patients were discharged 48 h after the infusion per national radiation safety regulations.

### Data Collection and Endpoints

Baseline demographics, mCRPC treatment history, and comorbidities (arterial hypertension and diabetes mellitus) were extracted from the LUMEN records. Serum creatinine was used to calculate eGFR, using the formula developed by the Chronic Kidney Disease Epidemiology Collaboration (5), for up to 24 mo. Endpoints were absolute and relative eGFR changes from baseline.

### Statistical Analysis

The overall longitudinal trajectory of eGFR was assessed using a univariate linear mixed-effects model (LMM) with time as the fixed effect and patient-specific random intercepts and slopes, assuming linear trajectories based on visual inspection (lmerTest version 3.1-3; R Project for Statistical Computing). To identify risk factors of renal function decline, a multivariable LMM was fitted to include interactions between time and potential risk factors (i.e., hypertension, diabetes, age ≥ 65 y, baseline eGFR, and cumulative activity). Generalized estimating equations with an exchangeable working correlation structure tested for shifts in chronic kidney disease (CKD) stages (geepack version 1.3.10; R Project for Statiscal Computing), with *P* values based on empiric standard errors. A *P* value of less than 0.05 was considered statistically significant. All analyses were performed using RStudio version 2025.05.1 + 513 (Posit Software).

## RESULTS

In total, 261 patients met the inclusion criteria. Key baseline characteristics are summarized in Table 1.

**TABLE 1.** Baseline Characteristics at Start of [^177^Lu]Lu-PSMA-617 Therapy (*n* = 261)

Characteristic	Value
Age (y)	
Mean ± SD	72.4 ± 7.96
Median	73.2 (67.1–78.3)
ECOG performance status	
0	125 (47.9)
1	93 (35.6)
2	33 (12.6)
3	4 (1.5)
Missing	6 (2.3)
No. cycles	
Median	4 (3–6)
Cumulative dose (GBq)	
Mean ± SD	28,800 ± 13,300
Median	25,100 (18,900–34,600)
Comorbidity	
* *Arterial hypertension	130 (49.8)
* *Diabetes mellitus	35 (13.4)
eGFR at baseline (mL/min/1.73 m^2^)	
Mean ± SD	81.4 ± 17.4
Median	85.2 (71.0–93.6)

IQR = interquartile range; ECOG = Eastern Cooperative Oncology Group.

Qualitative data are number and percentage. Continuous data are median and interquartile range.

### Longitudinal Outcomes

**FIGURE 2.**
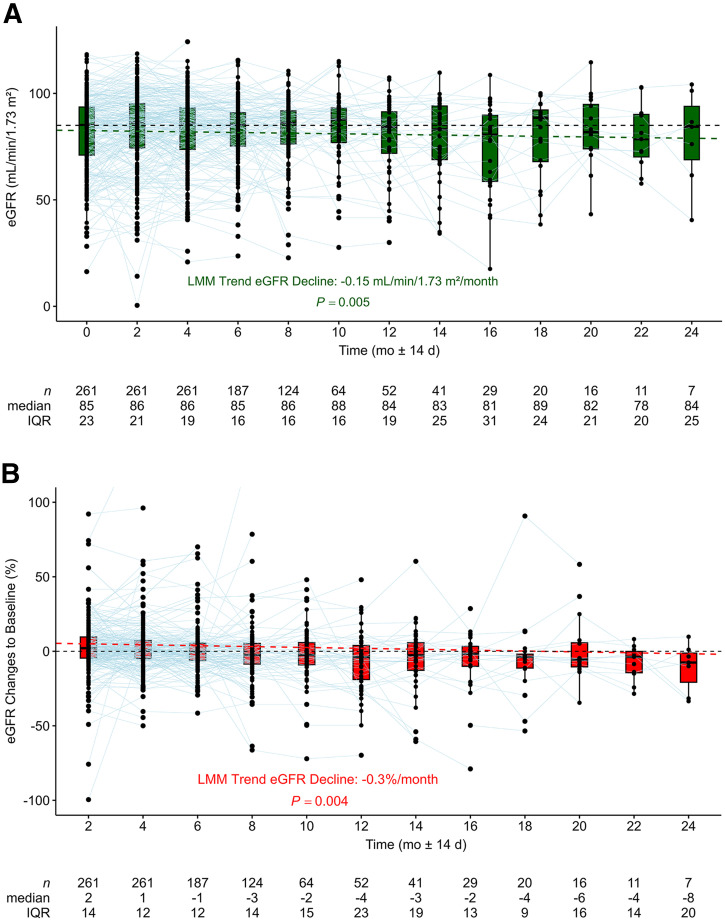
Spaghetti plots of individual longitudinal eGFR measurements in 2-mo intervals, given in absolute values (A) or relative to baseline (B). Box plots are given for each time point. LMM slope with significance indicator is provided. IQR = interquartile range.

The longitudinal analysis demonstrated a statistically significant, albeit small, decline over the 24-mo follow-up period (Fig. 2A). The LMM estimated an average decline of −0.15 mL/min/1.73 m^2^ per month (95% CI, −0.25 to −0.05; *P* = 0.005), translating to an estimated decline of −1.80 mL/min/1.73 m^2^ per year (95% CI, −3.03 to −0.57). The estimated relative decline was −0.3% per month (95% CI, −0.4 to −0.1; *P* = 0.004), resulting in a decline of −3.6% per year. To address potential survivorship bias, a sensitivity analysis restricted to patients with more than 12 mo of follow-up (*n* = 68) confirmed a statistically significant decline of −0.25 mL/min/1.73 m^2^ per month (95% CI, −0.40 to −0.10; *P* = 0.002).

### Dose Dependency

Patients receiving more than 6 cycles (*n* = 42) experienced a steeper decline in eGFR (−0.39 mL/min/1.73 m^2^ per month; 95% CI, −0.65 to −0.13; *P* = 0.006). This acceleration was confirmed by a significant dose–time interaction in the main model—higher cumulative activity accelerated eGFR loss by −0.008 mL/min/1.73 m^2^ per additional gigabecquerel and per month (95% CI, −0.015 to −0.001; *P* = 0.028), equating to approximately −0.1 mL/min/1.73 m^2^ (95% CI, −0.18 to −0.01) per year.

### CKD Staging

The proportion of patients with stage 3A CKD or higher shifted from 13.4% (baseline) to 14.3% (24 mo). The generalized estimating equation showed no significant risk for progressing to stage 3A CKD or higher over time (odds ratio, 1.02; 95% CI, 0.97–1.06; *P* = 0.518).

### Further Risk Factors

Although an age of 65 y or greater and hypertension were associated with lower baseline eGFR (*P* < 0.001 and *P* = 0.016, respectively), neither these nor diabetes significantly altered the rate of eGFR decline (*P* > 0.05; Table 2). A subgroup analysis of patients with stage 3A CKD or higher at baseline (*n* = 35) revealed a change in eGFR of 0.50 mL/min/1.73 m^2^ per month (95% CI, 0.03–0.97; *P* = 0.049).

**TABLE 2.** LMMs Comparing eGFR Changes by Risk Factors

Characteristic	*n*	Estimate (95% CI)*	*P*
Baseline eGFR (mL/min/1.73 m²)			
Reference group^†^	32	92.58 (88.03, 97.14)	
Hypertension	130	−4.91 (−8.91, −0.91)	0.016
Diabetes	35	0.63 (−5.11, 6.37)	0.829
Age ≥ 65 y	210	−9.16 (−14.01, −4.31)	<0.001
eGFR change per month (mL/min/1.73 m²)			
Reference group^†^	32	−0.16 (−0.47, 0.15)	
Hypertension	130	0.02 (−0.24, 0.27)	0.890
Diabetes	35	−0.08 (−0.44, 0.28)	0.670
Age ≥ 65 y	210	0.04 (−0.29, 0.36)	0.840

* Estimates and CIs for risk factors represent the difference compared with the reference group.

† Reference group consists of patients younger than 65 y without hypertension or diabetes.

## DISCUSSION

In this large real-world analysis, [^177^Lu]Lu-PSMA-617 therapy resulted in a statistically significant average annual decline of 1.8 mL/min/1.73 m^2^ in eGFR. Although this confirms a measurable renal impact, the magnitude is clinically modest for patients with mCRPC. An annual loss of less than 3 mL/min/1.73 m^2^ is generally not associated with increased mortality (6,7). For patients with late-stage cancer with limited life expectancy, this trajectory is unlikely to lead to symptomatic renal failure or dialysis.

Our findings align with renal safety data from the VISION and PSMAfore trials as well as smaller real-world series (1,8,9), but contrast with previous reports suggesting higher rates of a moderate eGFR decline in 33%–46% of patients (2,10). This discrepancy may stem from methodologic differences; our use of LMMs accounts for individual longitudinal trajectories, mitigating the bias of short-term physiologic fluctuations evident in single-point comparisons. The robustness of this methodology in an unselected cohort is evident when looking at the implausible result of increasing eGFR in our subgroup of patients with stage 3A or higher CKD. A regression-to-the-mean phenomenon has to be considered when selecting patients on the basis of extreme baseline variables, consistent with previous series demonstrating eGFR improvement during [^177^Lu]Lu-PSMA-617 therapy in patients with preexisting renal impairments (11).

Given these limitations in stratifying patients by baseline renal function, robust risk factors for treatment-associated eGFR decline remain a clinical need. In our analysis, those previously suggested (hypertension, diabetes, age ≥ 65 y (2)) did not seem to accelerate kidney function decline, suggesting the mechanism of radiotoxicity operates independently of vascular comorbidities. Instead, the significant interaction identified in our LMM analysis provides further confirmation and quantification of potential dose-dependent renal impairment, supporting causality and reinforcing the biologic plausibility of the observed functional effects (2,3,10).

Although the rate of eGFR decline may be negligible in late-stage mCRPC, a modest annual loss of 1.8 mL/min could translate into a clinically meaningful cumulative deficit (∼18 mL/min over a decade) when radiopharmaceutical therapy is applied earlier in the disease course, such as in hormone-sensitive settings, as in the UpFrontPSMA trial, where survival may exceed 5–10 y (12). Despite modest sample sizes, our cohort data indicate that the accelerated eGFR decline observed in long-term survivors (>12 mo), particularly in patients receiving more than 6 cycles, is consistent with cumulative renal toxicity, suggesting that this projection may be overly optimistic for these subgroups. Overall, these concerns emphasize the need for vigilance in patients undergoing extended treatment courses.

The strengths of our study include its large cohort size, drawn from a real-world registry at a single high-volume center, which ensures consistency in treatment protocols and patient management. Furthermore, the use of LMMs represents a robust statistical approach, allowing for the accurate modeling of individual patient trajectories while accounting for variability in follow-up schedules. Nevertheless, some limitations must be acknowledged. The retrospective design and use of clinically driven data pose risks of bias. A major limitation is the high attrition rate typical of patients with mCRPC; patients lost to follow-up because of death or clinical deterioration may have had worse renal trajectories than those who remained, introducing survivorship bias. Although our sensitivity analysis of long-term survivors supported the main findings, we cannot fully rule out that severe nephrotoxicity contributed to dropout in unobserved cases. We also did not account for potential nephrotoxic concomitant medications or other concurrent cancer therapies that could have influenced renal function.

## CONCLUSION

[^177^Lu]Lu-PSMA-617 demonstrates a favorable renal safety profile in real-world mCRPC practice, with a slow, predictable rate of function loss. However, evidence of dose-dependency suggests that, while the therapy is safe for current late-stage indications, rigorous long-term monitoring is essential as radiopharmaceutical therapy expands to patients with earlier stages of disease.

## DISCLOSURE

This publication was supported by the Open Access Publication Fund of the University of Bonn. Thomas Büttner reports speaker honoraria from Astellas Pharma. Philipp Krausewitz reports speaker honoraria from Novartis and Johnson and Johnson. No other potential conflict of interest relevant to this article was reported.

## Notes

KEY POINTS **QUESTION:** Is the use of [^177^Lu]Lu-PSMA-617 radiopharmaceutical therapy for mCRPC associated with significant, long-term kidney function decline? **PERTINENT FINDINGS:** In a large, real-world cohort of 261 patients with mCRPC, [^177^Lu]Lu-PSMA-617 treatment was associated with a statistically significant, but clinically modest, decline in renal function over 24 mo. The average eGFR decline was −1.80 mL/min/1.73 m^2^ per year. This annual reduction in eGFR is considered unlikely to be clinically relevant for this patient population. **IMPLICATIONS FOR PATIENT CARE:** [^177^Lu]Lu-PSMA-617 demonstrates a favorable renal safety profile in real-world mCRPC practice, with a slow, predictable rate of function loss. Although this is unlikely to be clinically relevant for late-stage mCRPC, the cumulative dose effect warrants careful monitoring in earlier disease stages.
